# Epidemiological urinalysis of children from kindergartens of Can Gio, Ho Chi Minh City - Vietnam

**DOI:** 10.1186/1471-2431-13-183

**Published:** 2013-11-11

**Authors:** Le Nhu Nguyet Dang, Thi Le Binh Doan, Ngoc Hue Doan, Thi Kim Hoa Pham, Françoise Smets, Mong Hiep Tran Thi, Françoise Janssen, Annie Robert

**Affiliations:** 1 Emergency Department, Children’s Hospital 2, Benh vien Nhi Dong 2, 14 Ly Tu Trong, District 1, Ho Chi Minh City, Vietnam; 2 Can Gio District Hospital, Ho Chi Minh City, Vietnam; 3 Center for Disease Control and Prevention of Can Gio, Ho Chi Minh City, Vietnam; 4 Département de Pédiatrie, Cliniques universitaires Saint-Luc, Université catholique de Louvain, Brussels, Belgium; 5 University of Medicine Pham Ngoc Thach, Ho Chi Minh City, Vietnam; 6 Université Libre de Bruxelles, Brussels, Belgium; 7 Institut de Recherche Expérimentale et Clinique, Pôle Epidémiologie et Biostatistique, School of Public Health, Université catholique de Louvain, Clos Chapelle-aux-Champs 30 box B1.30.13 BE-1200, Brussels, Belgium

**Keywords:** Chronic kidney disease, Dipstick, Urinary screening, Can Gio, Vietnam

## Abstract

**Background:**

Recent studies on Vietnamese children have shown that kidney diseases are not detected early enough to prevent chronic renal failure. The dipstick test is a simple and useful tool for detecting urinary abnormalities, especially in isolated or remote areas of Vietnam, where children have limited access to health care.

**Methods:**

This cross-sectional study was conducted in 2011 at seven kindergartens in Can Gio district, Ho Chi Minh City, Vietnam. Two thousand and twelve children, aged 3 to 5, were enrolled. Morning mid-stream urine samples were examined by dipstick. Children with abnormal findings were re-examined with a second dipstick and underwent further investigations.

**Results:**

Urinalysis was available for 1,032 boys and 980 girls. Mean age was 4.4 ± 0.8 years. Urinary abnormalities were detected in 108 (5.5%) of the subjects. Among them, nitrituria and leucocyturia accounted for more than 50%. Positive fractions of proteinuria, hematuria, nitrituria, leucocyturia, and combined nitrituria and leucocyturia after two dipsticks were 0.1%, 0.1%, 2%, 1% and 0.3%, respectively. Abnormal findings were more common in girls than boys (p < 0.001), and higher in communes with very low (< 50 persons/km^2^) population density (14.3% vs 4.1%, p < 0.001). A renal ultrasound detected four cases of hydronephrosis and one case of duplication of ureter.

**Conclusions:**

The prevalence of urinary abnormalities in asymptomatic children in South Vietnam demonstrates the need for hygiene education among parents. Training for dipstick usage for all medical staff at health stations, especially in remote areas and in places with very low population density, is also clearly necessary. Routine urinalysis can be set up if a close control is conducted at locations.

## Background

In Vietnam, as well as in many other developing countries, there is currently no national epidemiologic data on pediatric chronic renal failure (CRF). A retrospective study carried out by Mong Hiep *et al.*, from 2001 to 2005, in two departments of pediatric nephrology and in four departments of adult nephrology in Ho Chi Minh City (HCMC), reviewed 310 children aged less than 19 years who had been hospitalized for CRF. Median age was 14 years and 85% of patients were in end stage chronic kidney disease (CKD), compared to 14% in Belgian series [1,2]. Moreover, etiology of CKD in HCMC was different from those reported in developed countries. Mong Hiep pointed out the principal causes of CRF in these children: 30% were of glomerular origin, 20% of congenital and hereditary origin, and 50% were lacking information or a clinical investigation regarding etiology. In these clinical series from HCMC, only 27% received a substitutive treatment and the hospital death rate was 15%, quite higher than <1% in Belgian children with CRF [1,2]. Such data clearly suggest that the kidney diseases in Vietnamese children are not detected early enough to prevent complications. This was also evidenced by a median age at diagnosis that was much higher in the Vietnamese series (14 yrs) than in Belgian children (3 yrs).

The dipstick test is a basic method which is commonly used for screening renal diseases or urinary tract infections in asymptomatic subjects. A number of recommendations for urine screening have been published by the American Academy of Pediatrics (AAP) over the past 20 years. In 2007, the AAP recommended to discontinue urine screening in healthy children [3]. However, a screening program conducted in several Asian countries reported positive effects [4-7]. In Vietnam, where the health system is different from those in developed countries, little is known about urinary abnormalities in asymptomatic children, especially in isolated or remote areas of Vietnam, such as Can Gio district.

Can Gio is a rural coastal district of HCMC. The district is located 50 km from downtown HCMC. In 2009, the district had an area of 704 km^2^ and a population of 68,846 inhabitants corresponding to a density of 98 inhabitants/km^2^, compared to 11,826 inhabitants/km^2^ in urban districts of HCMC in 2009 [8]. Kinh is the major ethnic group, making up 80% of the population; the Khmer Krom and Cham people are the other ethnic groups of Can Gio. Mangrove forests are the dominant vegetation in Can Gio, which is separated at the center by a river where a ferry is used to cross it. Can Gio includes one town, Can Thanh, and six communes. One commune is an island that remains isolated and has many difficulties in terms of access and infrastructure. Can Gio has one district hospital and one Center for Disease Control and Prevention. The Can Gio hospital is located in Can Thanh Town and is far from the other communes. Patients' access to medical care is therefore inconvenient. The Center for Disease Control and Prevention manages seven health stations for many national health activities, e.g. the national immunization program, and a malnutrition prevention program. Beside these activities, the health stations of Can Gio supply a consultation service for citizens due to the district’s vast area, its non-concentrated population and its difficult access to a hospital. The head of each health station is a doctor (a medical school graduate) or a medical staff (a medical college graduate). The sparsity of tools available for diagnostic purposes at health stations is problematic. While modern means are difficult to acquire, the dipstick is a simple and cheap tool without any adverse effects.

The aim of our study was therefore to apply former AAP recommendations by setting up a systematic urinary screening program for children, as well as by implementing a health education campaign for parents in Can Gio in order to detect abnormalities and to prevent kidney diseases at an early stage.

## Methods

### Children

Between January and June 2011, a cross-sectional study was carried out in all kindergartens of Can Gio district, Ho Chi Minh city, Vietnam.

### Sampling method and data collection

The study was carried out with the support of the Children’s Hospital 2 (CH2) of HCMC (district 1) and with the help of the seven health stations of Can Gio. Seven dipsticks readers (DARA trademark, Linear Chemicals, Spain) were supplied to each health station near the kindergartens. The staff were previously trained to use the readers by technicians from CH2.

The screening was announced in advance to parents. An informed consent form and a clean urine container were supplied. Before the screening day, parents had been instructed on how to obtain the specimen.

At the day of screening, the first morning midstream urine sample that was collected at home in the supplied container and the signed informed consent form were brought to school in the early morning by parents.

The results were read that morning by a pediatrician and a technician from the CH2, with help of the health stations’ staff. Children with a positive dipstick were screened by a second dipstick the same day for confirmation. Those who were still positive after the second dipstick underwent renal ultrasound at Can Gio District Hospital. In children with positive leucocyte esterase and/or positive nitrites, another urine sample was collected for culture at the CH2. Children presenting proteinuria or hematuria were sent to CH2 for further investigations.

Dipsticks were supplied by Linear Chemicals (Spain) under the product name “Cromatest”. The dipstick contains 10 reagents: pH, gravity, protein, blood, glucose, leucocytes, nitrite, urobilinogen, bilirubin and ketons. Urinalysis was considered abnormal if at least one of the following findings were detected: 1) positive nitrites, 2) ≥ 25 white blood cells/μL (leucocyte esterase), 3) ≥ 10 red blood cells/μL, 4) proteinuria ≥ 150 mg/dL.

### Ethical considerations

Standards of ethics for studies conducted in Vietnam were respected. The study protocol was approved by the Ethical Committee of the HCMC Health Administration and by the Ethical Committee of Children’s Hospital 2 in HCMC. Informed consent was obtained from parents of all children involved in the study, with standard assurances of confidentiality.

### Statistical analysis

Data were analyzed using SPSS 20. Data are expressed as a number and percentage or as the mean and standard deviation. Proportions were compared using Chi square tests. A p-value lower than 0.05 was considered significant.

## Results

Can Gio communes’ characteristics and the number of children are presented in Table 1, by order of population density. Out of the 2,365 children aged 3 to 5 in Can Gio, 2,012 (85%) were screened with a first dipstick. Children who were not included in this screening (“Missing Children”) were mainly 3 years of age, and this was the case across all communes. Out of 449 (22.3%) positive dipsticks, 55 (12%) were not confirmed by a second dipstick because they were absent from class on the day of investigation or because we could not collect their urine sample. The remaining number of fully screened children was therefore 1,957 (83%). The mean age was 4.4 ± 0.8 years; five year-old children made up 53.1% of the population and the boy/girl ratio was 1032/980. The Kinh ethnicity account for 99.5% (1822/1831).

**Table 1:** Characteristics of communes and distribution of children aged 3 to 5 in Can Gio district

**Commune**	**Surface (km**^**2**^**)**	**Population (persons)**	**Population density (persons/km**^**2**^**)**	**Number of children aged 3-5**			
				**3 yrs**	**4 yrs**	**5 yrs**	**Total**
Can Thanh town	24.09	12,164	505	178	157	185	520
Binh Khanh	43.45	16,289	375	136	215	279	630
An Thoi Dong	103.72	13,519	131	57	98	265	420
Long Hoa	133.00	10,194	77	70	77	133	280
Tam Thon Hiep	110.38	6,227	56	41	46	93	180
Ly Nhon	158.16	5,923	37	45	46	104	195
Thanh An island	131.42	4,530	35	39	47	54	140
**Total**	**704.22**	**68,846**	**98**	**566**	**686**	**1113**	**2365**

There were 108 children (5.5%) with positive findings after 2 dipsticks out of 1,957 children screened at all seven kindergartens. The percentage of children who had at least one positive parameter was 22.3% at the first screening, and 27.4% (108/394) of them had urinary abnormalities at the second screening. After two tests, the overall percentage of children with positive results was the highest at Ly Nhon commune, followed by Thanh An commune, with 16.0% and 11.9%, respectively. These two communes also have the lowest population density. An Thoi Dong had the lowest percentage of positive dipsticks, at 1.1%. When comparing communes with less than 50 persons/km^2^ to communes with more than 50 persons/km^2^, positive findings were significantly higher (40/280 = 14.3% versus 68/1766 = 4.1%, p < 0.001) in low population density communes (Table 2).

**Table 2:** Sample of children from Can Gio and percentage of children with positive findings

**Commune**	**Number of missing children**	**Number of enrolled children**				**Number of positive findings**			Positive% (*)
		**3 yrs**	**4 yrs**	**5 yrs**	**Total**	**3 yrs**	**4 yrs**	**5 yrs**	
	**n (%)**	**n (%)**	**n (%)**	**n (%)**	**n (%)**				
Can Thanh town	112 (22%)	108 (61%)	127 (81%)	173 (94%)	408 (78%)	8	2	4	3.4
Binh Khanh	44 (7%)	113 (83%)	201 (93%)	272 (97%)	586 (93%)	6	11	20	6.6
An Thoi Dong	61 (15%)	18 (32%)	83 (85%)	258 (97%)	359 (85%)	0	2	2	1.1
Long Hoa	46 (16%)	42 (60%)	63 (82%)	129 (97%)	234 (84%)	3	3	4	4.4
Tam Thon Hiep	40 (22%)	12 (29%)	38 (83%)	90 (97%)	140 (78%)	0	1	2	2.2
Ly Nhon	29 (15%)	29 (64%)	41 (89%)	96 (92%)	166 (85%)	3	10	13	16.0
Thanh An island	21 (15%)	29 (74%)	40 (85%)	50 (93%)	119 (85%)	3	6	5	11.9
**Total**	**353**	**351**	**593**	**1068**	**2012**	**23**	**35**	**50**	**5.5**
	**(15%)**	**(62%)**	**(86%)**	**(96%)**	**(85%)**	**(6.6%)**	**(5.9%)**	**(4.7%)**	

*Actual percentage = Number of positive cases/Actual number of 2-dipstick urinalyses performed × 100.

**Figure 1:**
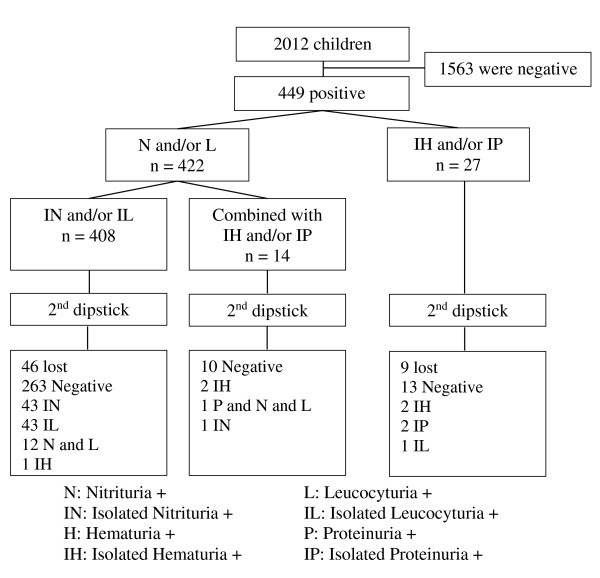
Urinary abnormalities among asymptomatic children in Can Gio after screening by two dipsticks.

When stratified by group of components, 408 children had isolated nitrituria (IN) and/or isolated leucocyturia (IL). Fourteen children had a combination of nitrituria (N) and leucocyturia (L), with or without isolated proteinuria (IP) and/or isolated hematuria (IH). Twenty-seven had IP and/or IH at the first dipstick. At the end of the second screening, there were 44 children with IN, 44 with IL, 12 with combined N and L, five with IH and 2 with IP, and one with combined P, N and L (Figure 1).

**Figure 2:**
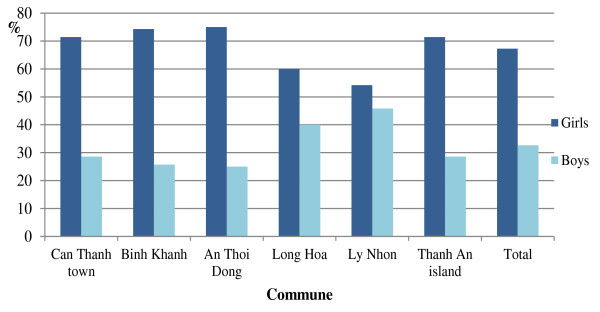
Positive nitrituria and/or leucocyturia in girls and boys within each commune of Can Gio having positive results.

**Figure 3:**
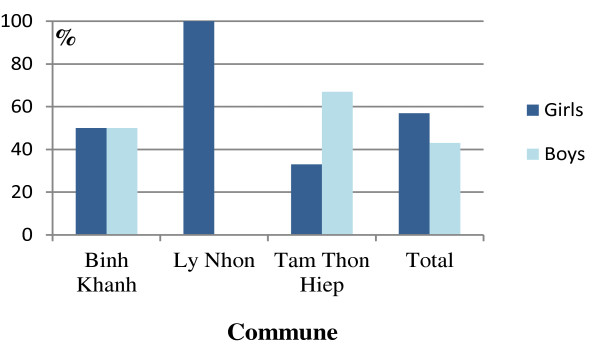
Positive proteinuria and/or hematuria in girls and boys within each commune of Can Gio having positive results.

In total, girls had more abnormal results than boys (72/108 and 36/108, p < 0.001). The differences between boys and girls in nitrituria and/or leucocyturia were significant (p < 0.001). No significant difference was found between the three age groups (Figures 2 and 3).

Additional file 1: Table S1 illustrates in detail the differences between the first dipstick and the second dipstick results. The total number of children checked by two dipsticks was 394. Overall, for the first and second dipstick, nitrite was the most common positive result (300/449 and 39/108, respectively), followed by leucocyte esterase (83/449 and 19/108, respectively). Of the 108 positive cases at the second test, nitrituria and leucocyturia account for more than 50%. The positive fractions of proteinuria, hematuria, nitrituria, leucocyturia, and combined nitrituria and leucocyturia after two dipsticks were 0.1% (2/1957), 0.1% (2/1957), 2% (39/1957), 1% (19/1957) and 0.3% (5/1957), respectively.

One hundred and two samples of urine were cultured at CH2, of which five samples presented one single type of bacteria. However, all of them had < 10^5^ colony forming units per mL. Two presented with *E. coli*, one with *E. faecalus*, one with *Negative-coagulase staphylococcus* and one with *Proteus*.

A renal ultrasound was performed at Can Gio, detecting 5/99 cases with the following: hydronephrosis (code N13.30 in ICD-10) in four cases and duplication of ureter (code Q62.5 in ICD-10) in one case. Four out of five cases with an abnormal ultrasound had a negative urine culture (Table 3).

**Table 3:** Abnormalities after work with urine culture and ultrasound in children with two positive dipsticks

**Case**	**Sex**	**Age**	**1st dipstick**	**2nd dipstick**	**Urine culture**	**Ultrasound**
1	M	3	Nitrituria	Nitrituria	*E. Coli*	Normal
2	F	5	Leucocyturia	Leucocyturia	*Proteus*	Normal
3	M	4	Nitrituria	Nitrituria	*Enterococcus faecalis*	Normal
4	M	5	Leucocyturia	Leucocyturia	*E. Coli*	Normal
5	F	5	Nitrituria	Leucocyturia	*Negative-coagulase staphylococcus*	Hydronephrosis
6	F	3	Nitrituria	Nitrituria + Leucocyturia	Negative	Hydronephrosis
7	M	5	Nitrituria	Leucocyturia	Negative	Duplication of ureter
8	M	4	Nitrituria	Nitrituria	Negative	Hydronephrosis
9	F	4	Nitrituria	Leucocyturia	Negatvie	Hydronephrosis

## Discussion

A urinary screening program is recommended as a fundamental element for decreasing the incidence of CKD [9]. However, the concern regarding cost-effectiveness was mentioned in studies of European and North American authors [10-12]. The retrospective study on cost-effectiveness conducted in children with hematuria and proteinuria, and published in Pediatrics 2010, supports the change in the AAP guidelines [10]. Nevertheless, according to the authors, this analysis was limited by several factors. In Asia, Japan was the first country to conduct a national screening program for children aged 6 to 14 in 1973 [6,13,14]. After that, other countries, like Taiwan, Korea, Malaysia and Singapore, have established screening programs. In these different studies, the collected urine was tested by a dipstick [4-7,13,15]. In Vietnam, the health care system is hardly making progress; children are not regularly followed by a doctor every 2 years like in the United States or in Europe. Moreover, doctors are not available at all health centers, particularly pediatricians. Can Gio is a typical example where the children do not maximally benefit from medical care. Therefore, implementing urinary screening programs should be considered in remote locations like Can Gio.

In Vietnam, to our knowledge, this is the first report about urinary screening in a large population of asymptomatic children. Dipsticks were used with the aims of searching proteinuria and/or proteinuria with hematuria for the detection of a nephropathy, and of searching proteinuria and/or leukocyturia or nitrituria in order to find partially treated pyelonephritis because in remote areas of Vietnam, there is no pediatrician and nearly all children presenting with fever are treated with antibiotics.

Vietnam is a developing country where little is known about the prevalence of urinary abnormalities in asymptomatic children. The prevalence of abnormalities has varied among different authors in different regions all over the world. Our prevalence in the first screening (22.3%) and the overall prevalence (5.5%) were higher than those reported in Nepalese, Lebanese and Malaysian studies [16-18]. In contrast, a higher prevalence has been reported in Nigeria by Akor *et al.*[19]. This variation may be due to different populations, different socioeconomic statuses and the prevalence of renal diseases within these populations.

While there were no differences between age groups, significant differences in the number of positive urinalyses across communes were found. Depending on the component, a positive dipstick can be the result of exercise, vaginal contamination, wrong sampling method, exposure of reagent strip to air, or consuming vitamin C or foods with high vitamin C content. The same brand of reagent strip, preserved in the same conditions, was used to test all children. Our results may therefore be explained by the special context of Can Gio. In particular, Thanh An and Ly Nhon are the two poorest communes in Can Gio, where the hygiene conditions are not good, fresh water is inadequate and the people have a low socioeconomic status. There is therefore a possibility of contamination and wrong sampling method that must be considered. This can also be evidenced more clearly by the prevalence of each separate component; the prevalence of nitrituria, leucocyturia and the percentage of false positives were high. As one can see, a supply of fresh water and hygiene education for parents and teachers in Can Gio are essential. In addition, a similar study in a population living in an urban area of HCMC is necessary for comparison and a subjective evaluation.

In our study, girls had a positive dipstick more frequently than boys. A similar result has been reported in other studies [13,15,17,19] whereas no gender difference was found in the Bakr *et al.*’s study [20]. This higher positive proportion included a predominance of nitrituria and leucocyturia. There was a significant difference for these two parameters between girls and boys in our results. Girls have short urethra, which facilitates ascending bacterial infection. Moreover, sampling is more difficult in girls than in boys.

Isolated nitrituria and isolated leucocyturia were the most two common abnormalities in our study. This is different from the findings of African authors [19]. As Vietnam is a tropical country, our result suggests an important prevalence of urinary tract infection (UTI) among these children. The exact prevalence of UTI is unknown for many reasons. Firstly, the signs are not specific. Secondly, there is the possibility of using the wrong technique for collecting urine in young children. Thirdly, antibiotics are often proposed to a febrile child and the urinary tract infection may be suppressed as a consequence. Two meta-analyses have shown a sensitivity of 0.88 for the presence of either leucocyte esterase or nitrite [21,22]. In contrast, the urine culture of 102 samples at CH2 in HCMC revealed no UTI because the samples had <10^5^ colony forming units per mL (CFU/mL) of one single type bacteria growing. All these cases with asymptomatic bacteriuria should not be treated with antibiotics nor with prophylactic antibiotics [23]. In Can Gio, children presenting with fever are commonly treated directly with antibiotics by physicians, without microbiological evidence, due to the lack of a paediatrician and the lack of a microbiology laboratory. Consequently, setting up a microbiology laboratory in Can Gio was deemed necessary in order to confirm diagnoses and to improve the treatment of UTI in children. This is why we have introduced a new protocol at the Can Gio District Hospital requesting a dipstick for each child presenting with fever and without any evidence of an infection focus; if leukocyturia and/or nitrituria is found, a urinary culture should be performed to search for bacteria. To follow this protocol, we have implemented a microbiology laboratory at the Can Gio District Hospital, with the help and the support of microbiologists from the Children’s Hospital 2. With the emergence of resistant bacteria worldwide, treatment with antibiotics based on nitrituria and/or leucocyturia before establishing the diagnosis by urine culture is not recommended, especially in a country with many infectious diseases like Vietnam.

The epidemiological study of bacteriuria in infants of a Swedish cohort reported positivity in 2.5% of boys and 0.9% of girls, and no major malformations were found after urography [23]. Westwood *et al.* concluded that there was no evidence to support the clinical effectiveness of routine investigation of children with confirmed UTI [24]. In our study concerning asymptomatic children, the five subjects with a positive urine culture for bacteria were investigated with a renal ultrasound; four of them were normal. The urography was therefore arguably not necessary. However, current recommendations for evaluation of UTI include performing an ultrasound and a voiding cystourethrogram after the first UTI. Searching for other modifiable host factors, such as voiding dysfunction or constipation, is crucial following the documentation of a UTI [25,26]. Hence, the appearance of any symptoms suggestive of a UTI in children should lead to a complete assessment, especially because vesicoureteral reflux is proved to be associated with recurrent infections [27,28]. Of course, vesicoureteral reflux is only interesting as a cause of UTIs in the youngest children or in those with recurrent pyelonephritis. Otherwise factors related to bladder function (including constipation) are much more important. This is why information campaigns were conducted in Can Gio for parents and educators on voiding disorders in children, pointing the importance of hygiene and hydration.

Hematuria and/or proteinuria are the aim of most urinary screenings among asymptomatic children because the finding of both hematuria and proteinuria may suggest the presence of an underlying renal disease. In our study, there were no cases of combined hematuria and proteinuria, although this number varies from 0.03% to 2.3% in other countries [5,7,13,18]. The number of children with isolated proteinuria was 0.1% at the second screening. Our prevalence was equivalent to data of Tokyo, Lebanon and Egypt, which were 0.8%, 0.1% and 1.2%, respectively [13,17,20]. We first used morning urine samples to exclude orthostatic proteinuria as a cause of false positive proteinuria. A very high urine pH may also be a cause of false positive proteinuria. This may explain why we had 7/10 cases with a negative protein at second dipstick. Renal biopsy and rigourous follow-up have allowed early intervention in selected cases, as evidenced by Japanese and Taiwanese populations [5,13]. In our cross-sectional study, one limitation was that we could not identify the underlying pathology of the two cases with isolated proteinuria because parents refused to continue the study.

The 0.1% prevalence of isolated hematuria in our study is lower than the 1.5% from previous studies [17,19] and higher than prevalence of Nepal, Malaysia Japan and Korea [13,15,16,18]. Only persistent hematuria reveals the presence of kidney diseases, of which the main causes are lupus nephritis and IgA nephropathy. Two children with IH in our study were directed to health stations for follow-up. Of the seven cases with positive hematuria on the first dipstick, five were negative on the second one. This may be due to contaminated urine specimens.

The number of false positives in our study was high for all four main components. Aside from the special conditions of Can Gio that we previously described, one element that must be considered is the population in our study’s young age, making it difficult to obtain mid-stream urine. According to Yanagihara *et al.*, in order to validate a screening program, attention should be paid to quality controls of the screening method, such as the selection of reagent strips, and the participants should be instructed to strictly adhere to the sampling method [6]. It is very difficult to instruct young children and parents, particularly in places with economical, health care and educational hardships. The disadvantage in the sample’s transportation is also an issue to take into consideration.

## Conclusions

This population-based study is the first report on urinary abnormalities in asymptomatic children aged 3 to 5 in South Vietnam. The high prevalence of nitrituria and leucocyturia demand hygiene education for parents, especially in remote areas. Knowledge about the usefulness of dipsticks is required for all medical staff at health stations: the use of dipsticks in district’s hospitals remains important when there is no pediatrician because children with fever are too quickly treated with antibiotics. Routine urinalysis can be part of preschool children medical examinations if a close control is conducted in all locations, especially in places with low socioeconomic status, with difficult access to health care, and with a very low population density.

## Abbreviations

AAP: American Academy of Pediatrics; CH2: Children’s hospital 2; CKD: Chronic kidney disease; CRF: Chronic renal failure; HCMC: Ho Chi Minh City; (I)H: (Isolated) Hematuria; (I)L: (Isolated) Leucocyturia; (I)N: (Isolated) Nitrituria; (I)P: (Isolated) Proteinuria.

## Competing interests

The authors declare that they have no competing interests.

## Authors’ contributions

DLNN participated in the design, carried out the study, performed the statistical analysis and drafted the manuscript. DNH and PTKH helped in data collection and report. DTLB helped in data analysis. SF provided advice in the design of the study and contributed to the manuscript revision. RA, JF and MHTT are head of the project, provided advice for the study design, the structure, the analytical strategy, and supervised the manuscript redaction. No author has any financial or private interest in this research project. All authors read and approved the final manuscript.

## Pre-publication history

The pre-publication history for this paper can be accessed here:

http://www.biomedcentral.com/1471-2431/13/183/prepub

## Supplementary Material

Additional file 1: Table S1 Cross table of dipstick components between the first and the second test. Click here for file
