# Supplementary material for: Epidemiological urinalysis of children from kindergartens of Can Gio, Ho Chi Minh City - Vietnam
Source: BMC Pediatr. 2013 Nov 11;13:183. doi: 10.1186/1471-2431-13-183 (PMC3829665; doi:10.1186/1471-2431-13-183)
Supplement: Additional file 1: Table S1 — Cross table of dipstick components between the first and the second test. [file 1471-2431-13-183-S1.docx]

**Table 3** Cross Table of Dipstick Components Between the First and the Second test

| **1st dipstick** | | | | | | | | | | | | | | | | | | | |
| --- | --- | --- | --- | --- | --- | --- | --- | --- | --- | --- | --- | --- | --- | --- | --- | --- | --- | --- | --- |
| **2nd dipstick** |  |  | P+H- | | | P-H+ | | | | P+H- | | | | P+H+ | | | | **Total** | |
|  |  |  | N+ | L+ | N+L+ | N+ | L+ | N+ L+ | N-L- | N+ | L+ | N+ L+ | N-L- | N+ | L+ | N+L+ | N-L- |  |  |
|  | P-H- | N+ | 39 | 3 | 1 |  |  |  |  |  | 1 |  |  |  |  |  |  | **44** |  |
|  |  | L+ | 21 | 19 | 3 |  |  |  |  |  |  |  | 1 |  |  |  |  | **44** |  |
|  |  | N+L+ | 4 | 3 | 5 |  |  |  |  |  |  |  |  |  |  |  |  | **12** |  |
|  |  | N-L- | 213 | 39 | 11 |  |  |  | 5 | 4 |  | 2 | 7 |  |  | 4 | 1 | **286** |  |
|  | P-H+ | N+ |  |  |  |  |  |  |  |  |  |  |  |  |  |  |  |  |  |
|  |  | L+ |  |  |  |  |  |  |  |  |  |  |  |  |  |  |  |  |  |
|  |  | N+L+ |  |  |  |  |  |  |  |  |  |  |  |  |  |  |  |  |  |
|  |  | N-L- | 1 |  |  | 1 | 1 |  | 2 |  |  |  |  |  |  |  |  | **5** |  |
|  | P+H- | N+ |  |  |  |  |  |  |  |  |  |  |  |  |  |  |  |  |  |
|  |  | L+ |  |  |  |  |  |  |  |  |  |  |  |  |  |  |  |  |  |
|  |  | N+L+ |  |  |  |  | 1 |  |  |  |  |  |  |  |  |  |  | **1** |  |
|  |  | N-L- |  |  |  |  |  |  |  |  |  |  | 2 |  |  |  |  | **2** |  |
|  | P+H+ | N+ |  |  |  |  |  |  |  |  |  |  |  |  |  |  |  |  |  |
|  |  | L+ |  |  |  |  |  |  |  |  |  |  |  |  |  |  |  |  |  |
|  |  | N+L+ |  |  |  |  |  |  |  |  |  |  |  |  |  |  |  |  |  |
|  |  | N-L- |  |  |  |  |  |  |  |  |  |  |  |  |  |  |  |  |  |
|  | No 2nd test | | 22 | 19 | 5 |  |  |  | 2 |  |  |  | 7 |  |  |  |  | **55** |  |
|  | **Total** | | **300** | **83** | **25** | **1** | **2** | **0** | **9** | **4** | **1** | **2** | **17** | **0** | **0** | **4** | **1** | **449** |  |

P Proteinuria, H Hematuria, N Nitrituria, L Leucocyturia.

(+) Positive (−) Negative.
